# Nanoemulsion-Based Hydrogels and Organogels Containing Propolis and Dexpanthenol: Preparation, Characterization, and Comparative Evaluation of Stability, Antimicrobial, and Cytotoxic Properties

**DOI:** 10.3390/gels8090578

**Published:** 2022-09-10

**Authors:** Rukiye Sevinç-Özakar, Emrah Seyret, Emrah Özakar, Mehmet Cemal Adıgüzel

**Affiliations:** 1 Department of Pharmaceutical Technology, Faculty of Pharmacy, Atatürk University, 25240 Erzurum, Turkey; 2 Faculty of Pharmacy, Atatürk University, 25240 Erzurum, Turkey; 3 Department of Microbiology, Faculty of Veterinary Medicine, Atatürk University, 25240 Erzurum, Turkey

**Keywords:** nanoemulsion, hydrogel, organogel, propolis, dexpanthenol, antimicrobial, cytotoxicity, stability

## Abstract

Recently, nanoemulsion-based gels have become very popular for dermal drug delivery, overcoming the disadvantages of conventional semi-solid drug forms. The aim of this study is to prepare and characterize nanoemulsion-based hydrogels and organogels containing combined propolis and dexpanthenol, and to compare their stability, antimicrobial, and cytotoxicity properties. Within the scope of characterization studies, organoleptic properties, drug content, morphology, pH, gel-sol conversion temperature, spreadability, viscosity, FT-IR, and release properties were evaluated in hydrogels and organogels. The characterization studies carried out were subjected to short-term stability evaluation at room temperature and refrigerator for 3 months. While no phase separation was observed in any of the formulations kept in the refrigerator, phase separation was observed in four formulations kept at room temperature. The release study successfully obtained an extended release for propolis and dexpanthenol. In the antimicrobial susceptibility study, Hydrogel 1 showed activity against *S. aureus*, while Organogel 1 showed activity against both *S. aureus* and *S. epidermidis*. In the cytotoxicity study against HDFa cells, both Hydrogel 1 and Organogel 1 were found to be nontoxic at low doses. These hydrogels and organogels, which contain propolis and dexpanthenol in combination for the first time, are promising systems that can be used in wound and burn models in the future.

## 1. Introduction

The skin is the largest organ of the human body. The skin has many functions, such as maintaining fluid balance, regulating body temperature, protecting against microorganisms, preventing microbial infections, and preventing the loss of all kinds of nutrients, water, electrolytes, and other substances in the body. At the same time, the skin is the largest part of the human body that comes into contact with the external environment or microorganisms. A wide variety of bacteria are found on the skin surface, including staphylococci, streptococci, candidiasis, and non-pathogenic mycobacteria. When the skin is damaged, it becomes vulnerable to infections from the external environment, and microorganisms can accumulate around the wound and cause infection. The skin, which is an obstacle to direct contact with the internal and external environment, is constantly exposed to many factors that can cause skin damage [1,2]. Apart from these, the skin serves as the primary target and major barrier for dermal/transdermal drug delivery. Due to its large, easily accessible surface area, it has gained great research interest as a non-invasive alternative route to traditional oral or injectable drug administration. Dermal/transdermal drug administration offers distinct advantages such as increasing the bioavailability of drugs damaged by the gastrointestinal environment and/or hepatic first-pass effects, transporting drugs at a constant rate over a long period of time, reducing side effects, and improving patient compliance. Despite all this, dermal/transdermal drug delivery is still an attractive and challenging issue [3]. Advances in modern technologies allow dermal/transdermal delivery of a large number of drugs, including conventional hydrophobic small molecule drugs, hydrophilic drugs, and macromolecules [4]. In recent years, there has been an increasing interest in colloidal drug delivery systems. In previous years, the focus of the application of nanocarriers has been primarily on parenteral and oral administration. However, nanocarriers applied to the skin offer many advantages. Nanocarriers can be used on the skin for both local action (dermal drug delivery) and systemic action (transdermal drug delivery) [5]. Nanocarriers not only provide direct contact of therapeutic agents with the stratum corneum and other extensions of the skin but also increase their physical and chemical stability by prolonging their persistence on the skin surface. Most conventional dermal treatments act by forming thin films on the skin surface, but using nanocarriers prevents the deposition of free drugs in these film layers. These film layers form a concentration gradient that advances therapeutic agents to the skin layers by diffusion. In recent years, different nanocarriers such as nanoemulsions, nanostructured lipid carriers, solid lipid nanoparticles, polymeric nanoparticles, liposomes, ethosomes, transferosomes, niosomes, aquasomes, and menthosomes have been extensively investigated to solve various skin problems [6]. Nanoemulsions are heterogeneous isotropic systems in which one liquid is dispersed in another liquid as nano-sized droplets. Droplet sizes can range from 20 to 500 nm. They are thermodynamically unstable and kinetically stable systems. Therefore, they need energy for their formation and surfactants and/or co-surfactants to provide colloidal stability [7,8]. The droplets of nanoemulsions, which have a higher surface area-to-volume ratio, act as a drug reservoir and ensure that the bioavailability of drugs is higher than conventional emulsion. Nanoemulsions show many important advantages such as optimum drug release, prolonged efficacy, low side effects, and drug protection from enzymatic or oxidative processes [9]. Nanoemulsions can be made into various dosage forms such as liquids, creams, sprays, gels, aerosols, and foams. There are application areas by oral, intravenous, intranasal, pulmonary, ocular, and topical routes [10].

Nanoemulgels are basically emulsion-containing topical gel formulations. Because nanoemulgels contain both nanoemulsion and gel base, that is, they exhibit dual character, they are among the suitable options as drug delivery systems. While the nanoemulsion in the structure of the nanoemulgel provides the protection of the active substance, the gel base provides thermodynamic stability to the nanoemulsion by increasing the viscosity of the aqueous phase. It has been suggested that some commercially available topical dosage forms have a low spreading coefficient compared to nanoemulgels, thus focusing on the application of nanoemulgels in the field of dermatology [11,12]. Hydrogels and organogels are among the different gel types in the literature. In general, hydrogels or organogels can be categorized based on the polarity of the external liquid component. As the external liquid component, hydrogels are prepared with water, while organogels are formulated using non-polar solvents such as hexane, isopropyl myristate, sunflower oil, corn oil, or others [13]. Hydrogels and organogels are semi-solid systems prepared from a polymer/gelling agent that can self-assemble and form a three-dimensional network structure. Chemical or physical cross-links that provide the network structure and physical integrity of the hydrogels render the hydrogels insoluble. Research on these systems has gained momentum in the last few years [14]. Hydrogels are three-dimensional polymer networks with a high-water content that can swell in aqueous solutions with mucoadhesive and bioadhesive properties and are excellent drug delivery systems. Conventional hydrogels are frequently used in tissue engineering, drug delivery, and other biomedical areas. Hydrogels have self-healing abilities even if their structure is damaged [15,16]. Polymers such as hydroxypropyl methylcellulose, polyvinyl alcohol, carbopol, poloxamer, and tragacanth gum are often used to prepare hydrogels [11]. Depending on the mechanism of creation of the three-dimensional gel skeleton, organogels are regarded as liquid-filled structures and solid fiber-based gels. The use of these organogels is increasing due to their easy preparation methods and long-term stability. They are thermoreversible and have the ability to contain both hydrophilic and hydrophobic compounds in their gel structure. This feature has also expanded the scope of organogels used as controlled drug delivery systems that can be taken by various routes of administration [13]. 

Propolis is the most well-known bee product after honey and is a resin mixture collected by Apis mellifera from different plants. There are more than 300 components in the content of propolis. In general, propolis consists of approximately 50% resin, 30% wax, 10% aromatic oils, 5% various organic compounds, and 5% pollen. Propolis is the most prosperous bee product in terms of polyphenol content [17]. Although propolis is not regarded as a therapeutic agent in traditional medicine, it is one of the few natural products that has long retained its popularity. It is widely used as an ingredient in cosmetic and pharmaceutical products such as anti-acne creams, face and body creams, ointments, lotions, and in various formulations for oral hygiene. Doctors used propolis during the Second World War (1939–1945) to treat wounds [18]. Propolis has anticancer, immunomodulatory, anti-inflammatory, antioxidant, and antimicrobial effects. Today, it is used especially for skin diseases (wounds, acne, warts, etc.), oral problems (gingivitis, denture adhesive, fungal infections), cardiovascular system diseases (atherosclerosis, hypertension), diabetes, and reproductive health problems [19].

Dexpanthenol, a component of coenzyme A, is an alcoholic analog of pantothenic acid (vitamin B5). Inside the body, dexpanthenol is converted into pantothenic acid, its active form necessary for epithelial cells. Unlike pantothenic acid, dexpanthenol is better absorbed through the skin and binds to tissues with high penetration. Dexpanthenol is freely soluble in water and alcohol and practically insoluble in oils. It provides good skin penetration when applied with topical formulations such as water-in-oil emulsions of dexpanthenol. Although the exact mechanism is unknown, topical dexpanthenol acts as a moisturizer that improves stratum corneum hydration, reduces transepidermal water loss, and maintains skin softness and elasticity. Its moisturizing effect is related to its hygroscopic property. The most prominent effects of dexpanthenol include stimulation of epithelialization and prevention of pruritus. Still, positive results have been observed in patients treated for skin transplants, wounds, burns, and different dermatoses. Studies have shown that skin care with dexpanthenol for more than 3–4 weeks significantly improves the symptoms of skin irritations such as dryness, roughness, itching, erythema, and cracks. The proliferation of fibroblasts is an essential factor in wound healing, and in vitro experiments with dexpanthenol have shown that it increases the proliferation of human fibroblasts [20,21].

In this study, in line with our purpose, nanoemulsion, hydrogel, and organogel formulations containing dexpanthenol in the outer phase and oil-based propolis in the inner phase were developed to be used in future wound model studies and evaluated in terms of characterization, stability, antimicrobial activity, and cytotoxicity. In the literature search, we see that there is neither nanoemulsion, gel, nor any other formulation containing these two substances. In this study, the combination of propolis and dexpanthenol was formulated and evaluated for the first time. However, in the literature search, it is seen that there is only one article about the pure use of dexpanthenol and propolis extract with antibiotics, and it has nothing to do with our study [22].

## 2. Results and Discussion

We would like to emphasize that propolis and dexpanthenol were formulated in combination for the first time in our study. Recently, especially conventional gels have been replaced by hydrogels, organogels, and other gel types. In this study, we wanted to focus on hydrogels and organogels in particular. We frequently see in the literature that hydrogels are water-based and organogels are often oil-based. Both formulation types have their own advantages. We successfully supported the characterization studies of the formulations we prepared with the short-term stability results we obtained by keeping them at room temperature and refrigerator conditions for 3 months. At the same time, antimicrobial and cytotoxicity studies gave us guiding results. Below are the comparative data we found and their discussion. In our study, the propolis oily extract was then expressed as propolis.

### 2.1. Development of Quantification Method for Propolis and Dexpanthenol

**Figure 1:**
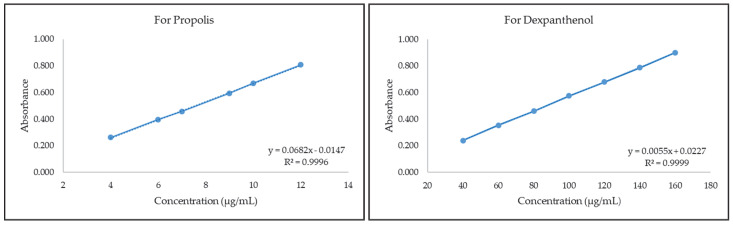
Calibration curves and equations of propolis and dexpanthenol.

In our study, the method for quantification of propolis oily extract standardized in terms of polyphenols (>30 mg/mL, HPLC-ESI-MS) was successfully developed with a UV-VIS spectrophotometer in ethanol. Validation studies were carried out at this wavelength since propolis gave a max peak at 290 nm. The LOD and LOQ values we obtained in our study were found to be 0.819 and 2.482 µg/mL, respectively. Intraday and interday accuracy and precision values were found below 2% according to the ICH Q2A. When the spectrophotometric studies of propolis in the literature are examined, it is seen that the maximum wavelength of ethanol extracts of propolis is around 290 nm, and it provides a broad spectrum. In ethanol extracts of propolis, especially phenolic compounds predominate in the formation of peaks. Similar peaks were obtained in the detailed studies of Maldonado et al. and Fabris et al., and it was emphasized that these peaks were caused by the phenolic compounds found in the structures of propolis extracts [23,24]. For dexpanthenol, the UV-VIS spectrophotometric method was successfully developed, and the quantification method was validated. The LOD and LOQ values we obtained in our study were found to be 4.098 and 12.419 µg/mL, respectively. Intraday and interday accuracy and precision values were found below 2% according to the ICH Q2A. There are not many UV-VIS spectrophotometric methods for dexpanthenol in the literature. The method of Poláček et al. was applied with minor modifications, but depending on the modifications, our wavelength (211 nm) that provided the max absorbance was found to be different [25]. The calibration curves and equations of propolis and dexpanthenol are given in Figure 1 below.

### 2.2. Preparation of Nanoemulsions

The oil phase, water phase, and surfactant/cosurfactant are needed to form nanoemulsions. Surfactants are adsorbed on the oil/water interface, causing a decrease in surface tension, resulting in a reduction of droplet size and formation of nanoemulsions. In most cases, the use of surfactants alone is insufficient to reduce the interfacial tension required for nanoemulsion formation, so a co-surfactant is used. Therefore, the use of dual surfactants in the preparation of nanoemulsions provides the ultra-low interfacial tension required to reduce the droplet size to the nanometer range [26]. The dual use of Tweens and Spans in the preparation of nanoemulsions is quite common in the literature [27,28]. The fact that Tweens are hydrophilic and Spans are lipophilic is a great advantage in terms of bringing the hydrophilic–lipophilic balance to the desired level [29]. Since propolis is a standardized extract prepared in sunflower oil, 758.33 mg was weighed directly for each formulation, making the dose equivalent to 25 mg on polyphenols. Dexpanthenol, on the other hand, took place in the outer phase of nanoemulsions due to its water-soluble properties. All our formulations have been successfully prepared as milky, uniform nanoemulsions without phase separation by high-energy ultrasonication technique. The following table contains formulation components and amounts (Table 1).

### 2.3. Characterization of Nanoemulsions

Droplet size, zeta potential, and polydispersity index values affect many properties of nanoemulsions, especially long-term physical stability and bioavailability [8]. As a result of the analysis, the droplet size, polydispersity index, and zeta potential values of the nanoemulsions were found in the range of 166.0 ± 0.97–221.6 ± 4.71 nm, 0.149 ± 0.002–0.216 ± 0.005, and −29.8 ± 0.45–−40.0 ± 1.42 mV, respectively, as seen in Table 2. The uniformity of the droplet size distribution is measured by the polydispersity index, and generally, the nanoemulsion is called ‘monodisperse’ if the polydispersity index is < 0.25 [30]. Nanoemulsions are monodisperse, as all polydispersity index values are less than 0.25. When statistically evaluated, Nanoemulsion 2 and Nanoemulsion 3 were significantly different (*p* < 0.05) according to the droplet sizes of other nanoemulsions, while Nanoemulsion 1 was found to significantly different (*p* < 0.05) from only Nanoemulsion 2 and Nanoemulsion 3. Again, Nanoemulsion 5 was found to be significantly different (*p* < 0.05) from the others except for Nanoemulsion 1. The zeta potential is the most crucial parameter that determines the surface charge of nanoemulsion droplets. For long-term stability in nanoemulsions, a zeta potential value of more than ± 30 mV is desired [8]. The zeta potential values of our nanoemulsions are all greater than 30 mV and are negative. It is also stated in the literature that a negative charge provides more electrochemical stability than a positive charge [31]. When statistically evaluated, Nanoemulsion 6 was significantly different (*p* < 0.05) according to the zeta potentials of other nanoemulsions, while Nanoemulsion 1 was found to be significantly different (*p* < 0.05) from only Nanoemulsion 6. In general, we saw that the droplet sizes of the nanoemulsions were smaller when the surfactant usage ratios are 2:1 instead of 1:1. At the same time, the zeta potentials were higher when the surfactant usage ratios were 2:1 instead of 1:1. Here, we can say that the usage rates and amounts of surfactants (200 mg or 300 mg) have significant effects on droplet size and zeta potential. Similar results were observed in the study of Algahtani et al. [32].

**Figure 2:**
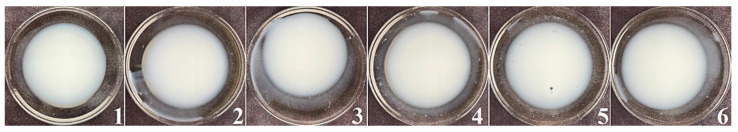
The nanoemulsion images obtained by dilution with ultrapure water. The numbers are given in order of nanoemulsions.

The type of determination of nanoemulsions was determined by two techniques: electrical conductivity measurement and dilution. When conductivity values were examined, it was seen that all results were above 7.20 ± 0.50 µS/cm in Table 2. The effect of surfactant ratios and amounts is also evident here, and as the amount increased, the conductivity decreased very little. Similar results were obtained in the studies of Špaglová et al. and Hasssanzadeh et al. [33,34]. Again, homogeneous, white, non-phase separation nanoemulsions were obtained in the dilution technique, which indicates the miscibility of the nanoemulsion with ultrapure water. The images obtained by dilution with ultrapure water are given in Figure 2. These results support the conductivity results, and we can easily say that the outer phase for the prepared nanoemulsions is water. Although the skin has a pH of 5.5, topical preparations with a pH between 4 and 7 are physiologically harmless and non-irritating [33]. The pH values of all nanoemulsions prepared are in this range, but the most important thing is the pH values of the final product, hydrogels, and organogels.

### 2.4. Preparation of Hydrogels and Organogels

**Figure 3:**
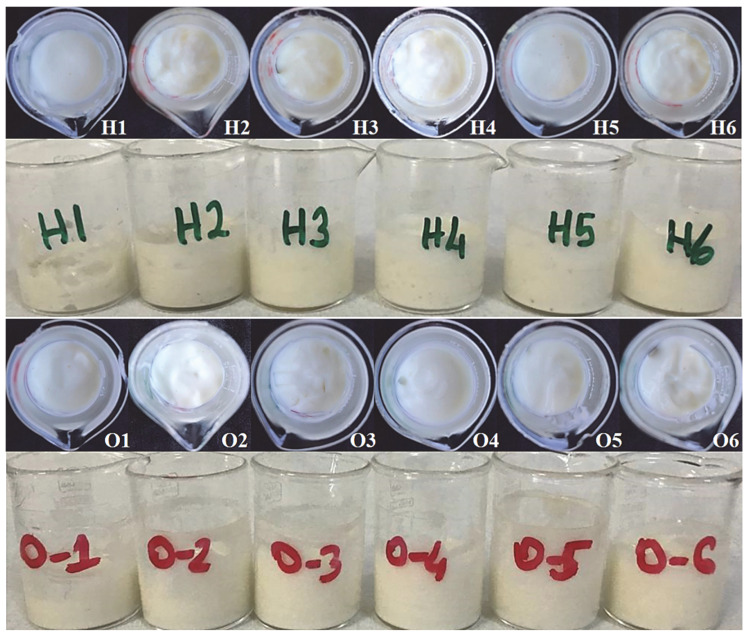
Top and side photographs of prepared hydrogels and organogels. Hydrogels are coded with letter H and organogels with the letter O.

Images of freshly prepared hydrogels and organogels are given in Figure 3 below. All formulations were successfully prepared in a smooth and homogeneous manner. Hydrogels and organogels are coded according to the nanoemulsion numbers they contain. In other words, the hydrogel containing “Nanoemulsion 1” was coded as “Hydrogel 1”, and the organogel containing “Nanoemulsion 1” was coded as “Organogel 1”.

### 2.5. Characterization Studies of Hydrogels and Organogels

#### 2.5.1. Organoleptic Characteristics

Freshly prepared hydrogels and organogels were all initially pale yellow to white in color and had a characteristic propolis odor. All of them were homogeneous and there were no phase separations as seen in Figure 3 above. In order to see the changes in their physical stability, they were kept at room temperature (24 ± 2 °C) and also in the refrigerator (4 ± 2 °C) for 3 months. Table 3 shows the phase separations and changes in colors after 3 months of storage. There was no change in their odor after 3 months. However, phase separations with liquefaction were observed in Hydrogels 4, 5, and 6, and Organogel 6. Slight color changes were also observed in these formulations with phase separation. Formulations with phase separation were not used in other analyses. When hydrogels and organogels are examined, different surfactants used in the formulations and their different amounts can be considered as the reason for the phase separation. In a study by Takamura et al., nanoemulsions were developed using different Tweens and Spans, and their stability was evaluated [29]. As a result of the evaluation, the researchers emphasized that the formulations in which Tween 20 and Span 80 were used together were not stable and the reason for this was the high difference (12.6) between the HLB values of Tween 20 and Span 80. The researchers also stated that when Span 20 and Tween 80 or Span 20 and Tween 40 are used together, formulations with high stability are obtained due to the low differences between HLB values (6.4 and 7.1, respectively). We think that the same situation applies to our formulations as well. Our hydrogels only contained surfactants from nanoemulsions, ase well as Span 20 and Tween 60 in organogels. These surfactants also supported the stability of the organogels, but only Organogel 1, with the lowest average HLB (8.6), had stability problems. That is, our results were similar to those of Takamura et al.

#### 2.5.2. TEM Images

**Figure 4:**
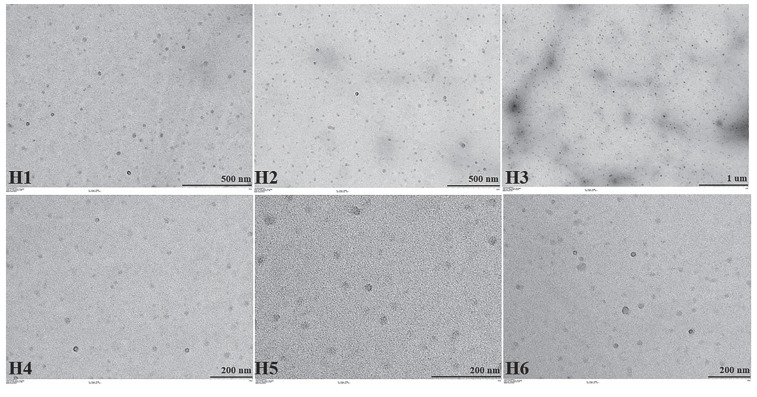

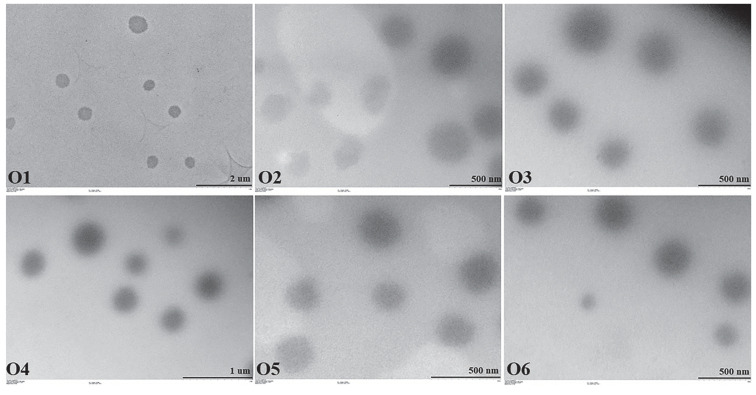
TEM images of hydrogels and organogels. Hydrogels are coded with letter H and organogels with the letter O.

TEM images of hydrogels and organogels were taken successfully and are shown in Figure 4 below. It is clearly seen in the images that the nanoemulsion droplets remain intact and maintain their sphericity.

#### 2.5.3. Drug Contents

The drug contents of hydrogels and organogels were successfully analyzed by UV-VIS spectrophotometer. The results obtained are given in Table 4 below. When all the results were evaluated, it was seen that the drug contents were between 98.15 ± 0.85–102.88 ± 0.30% and the standard deviation values were less than 5%. As everyone knows, in pharmacopoeias, it is requested that the deviation in drug contents should not be more than 5%, although there are exceptional cases. Our results did not exceed this limit and we also saw that the formulation components did not have a negative effect on propolis and dexpanthenol during 3 months of storage.

#### 2.5.4. pH and Gel-Sol Transition Temperature

The pH values of the formulations to be applied topically are very important in terms of compatibility with the skin, non-irritation, and non-allergenicity, and this is an issue that must be evaluated. Table 5 below shows the pH and gel-sol transition temperatures of the hydrogels and organogels in their fresh state and after 3 months of storage at room temperature and in the refrigerator. 

When all pH values were examined, it was seen that all results were between 4.35–6.54. In general, it was seen that the pH values decreased when nanoemulsions came together with hydrogels and organogels. However, the decrease in organogels was greater than in hydrogels. When the pH values of both freshly prepared hydrogels and organogels were compared with the values after 3 months of storage, it was seen that there was no excessive change in pH values in general. All pH values are in the range of 4–7. Although the skin had a pH of 5.5, topical preparations with a pH between 4 and 7 are physiologically harmless and non-irritating [33]. Therefore, prepared hydrogels and organogels are considered to be compatible with topical application. There are many studies in the literature that found similar results [35,36,37]. When the gel-sol transition temperature values were examined, quite high temperatures were observed in the range of 80–90 °C. These high-temperature values suggest that the prepared hydrogels and organogels were not immediately affected by small temperature changes and need to be exposed to very high temperatures to liquefy. When the 3-month data were also examined, there was not much change in gel-sol transition temperatures. Gel-sol transition temperature studies with hydrogels and organogels are very limited in the literature. Hatakeyama et al. found gel-sol transition temperatures in the range of 50–90 °C in their study using PVA hydrogel and DSC [38]. In the organogel study prepared by Gopalan et al., the researchers found gel-sol transition temperatures in the range of 42–50 °C [39].

#### 2.5.5. Spreadability and Viscosity Analysis

**Figure 5:**
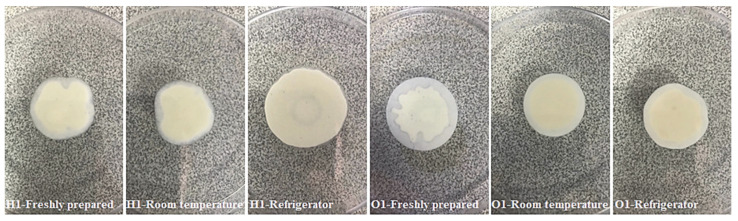
Sample spreadability images of hydrogels and organogels. Hydrogels are coded with letter H and organogels with the letter O.

The spreadability gives information about how easily topical drugs can be applied. Ease of application, especially on damaged skin, is a desirable feature. The higher the spreadability value, the easier the spreadability. The high degree of spreadability of the topical drugs ensures better patient compliance. Spreadability is also a parameter related to viscosity. High viscosity can also make spreadability difficult [40]. Sample spreadability images of hydrogels and organogels are given in Figure 5 below. In this study, it was obtained that the spreadabilities of hydrogels and organogels were in the range from 14.64 ± 0.08–30.93 ± 1.32 and 17.67 ± 0.13–23.97 ± 1.15 g·cm/s, respectively (Table 6). When examined in terms of diameter, it was seen that the results were in the range of 24.4 ± 0.1–51.6 ± 2.2 mm. When the freshly prepared hydrogels were statistically evaluated, Hydrogel 1 was significantly different (*p* < 0.05) from only Hydrogel 2, Hydrogel 4, and Hydrogel 6. Again, Organogel 1 was significantly different (*p* < 0.05) from only Organogel 2, Organogel 4, and Organogel 6. In the HPMC nanoemulgel study by Shetata et al., a diameter of 48.6 ± 2.9 mm was obtained and was interpreted as satisfactory spreadability [8]. Again, in the Carbopol 940 nanoemulgel study conducted by Arora et al., the spreadability results were found in the range of 5.5 ± 0.18–6.0 ± 0.54 g·cm/s [40]. Our results are well above these results, and it can be said that the spreadability values are quite ideal.

Viscosity is a crucial parameter in semi-solid formulations, especially regarding many properties such as flowability, spreadability, diffusion, and release of the active substance from the vehicle. Viscosity values of prepared hydrogels and organogels are given in Table 6. In general, the viscosity values are in the range of 143.9 ± 6.6–3690.7 ± 197.8 cP. It is clearly seen that the viscosity values of hydrogels are higher than those of organogels. Ultra-high viscosity NaCMC was used at a concentration of 1.6% in hydrogels and Carbopol 980 at a concentration of 0.4% in organogels. It is thought that this concentration difference also affects viscosity. The viscosity of the 1% aqueous dispersion of NaCMC at 25 °C is 1500–4500 mPa·s [41]. The viscosity of 0.5% aqueous dispersion of Carbopol 980 is 30,000–40,000 mPa·s [42]. Although there is such a viscosity difference between polymers, the concentration of these polymers in the final hydrogels and organogels was the determining factor. When we evaluate hydrogels and organogels in terms of viscosities after keeping them at room temperature and in the refrigerator for 3 months, a decrease is observed in the viscosity of those kept at room temperature. Looking at the phase separation test results, phase separation, and liquefaction, a decrease in viscosity was observed in some formulations. It is also thought that it would be more appropriate for stability to keep the hydrogels and organogels prepared from these results in the refrigerator instead of at room temperature. It is also thought that Organogel 4 and Organogel 5, which have the lowest viscosity values, will undergo phase separation in longer keeping times. When the freshly prepared and kept for 3 months in refrigerator hydrogels were statistically evaluated, Hydrogel 1 was significantly different (*p* < 0.05) from the other hydrogels. Again, Organogel 1 was significantly different (*p* < 0.05) from the others except for Organogel 6. Almostafa et al. found very high viscosity values of 15,245.0 ± 360.3 and 25,265.0 ± 400.2 cP in the gel and nanoemulgel, respectively, which were prepared with NaCMC at 2% concentration [35]. Compared to our study, it can be said that the spreadability of our formulations may be easier due to their lower viscosity. In addition, Shehata et al. HPMC at a 2% concentration was used in the gel and nanoemulgel, and it found very high viscosity values such as 11,580 ± 775.8 and 29,920 ± 1373.9 cP, respectively [8]. 

#### 2.5.6. FT-IR Analysis

**Figure 6:**
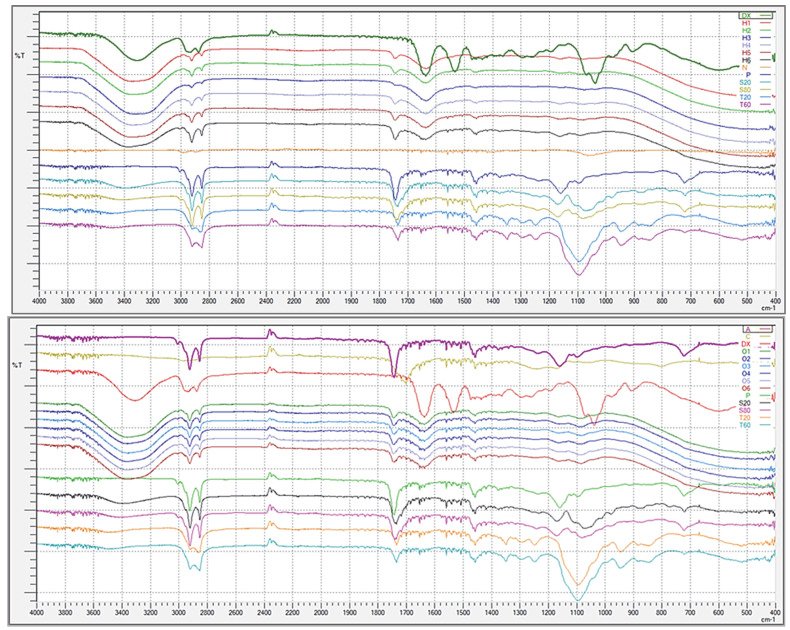
FT-IR spectra of hydrogels and organogels. Hydrogels are coded with letter H and organogels with the letter O. In addition, Dx: Dexpanthenol, N: NaCMC, P: Propolis, S20: Span 20, S80: Span 80, T20: Tween 80, T60: Tween 60, A: Sunflower oil, C: Carbopol 980.

The FT-IR spectra of the propolis, dexpanthenol, hydrogels, organogels, and all the excipients used in the nanoemulgels are given in Figure 6 below.

When the spectra of hydrogels and organogels were evaluated within themselves, it was seen that identical spectra were obtained. In particular, some specific peaks of Tweens and Spans (3000–2800, 1800–1700, 1500–1400 cm^−1^) were evident in the spectra of both hydrogels and organogels. Surfactant peaks were observed more (3000–2800, 1800–1400, 1150–1050 cm^−1^) in organogels due to the presence of additional surfactants apart from the surfactants coming from nanoemulsions. Based on these data, it can be said that the structures of surfactants in formulations remained intact. Specific peaks of propolis were seen at 3000–2800 2400–2300, 1800–1700, 1500–1400, 1200–1100, and 750–700 cm^−1^ intervals. Propolis is not a pure substance, on the contrary, it has a complex chemical composition. Its content may vary according to the region and climate from where it is obtained. The chemical composition of propolis can generally be divided into two groups: balsam (40–70%) and non-balm content (20–35%), which consists of many phenolics. This complex content allows for the formation of many peaks [43,44]. In particular, in the study of Svečnjak et al., all the peaks of propolis were explained in detail and are quite similar to the spectrum we obtained [44]. Additionally, specific sharp peaks of dexpanthenol were seen at 3000–2800, 2400–2300, 1700–1400, 1150–1000, and 950–850 cm^−1^ intervals. In the FT-IR analysis of Tuncay-Tanrıverdi et al. and Tamizi et al.’s study with dexpanthenol, peaks similar to the ones obtained in our analysis were obtained [45,46]. The fact that the sharp peaks of propolis and dexpanthenol appear suppressed in the spectra of hydrogels and organogels means that these two active substances are confined to the formulations. It is normal that the peaks of the active ingredients in the formulation are suppressed compared to their pure form and there are many similar studies in the literature [47,48,49].

#### 2.5.7. In Vitro Release and Release Kinetics

**Figure 7:**
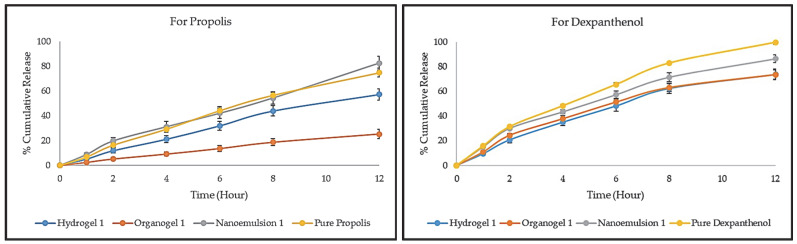
Release profile of propolis and dexpanthenol (mean ± SD).

The release studies of propolis and dexpanthenol from hydrogels, organogels, and nanoemulsions was successfully carried out using the dialysis bag method for 12 h. The release profiles are given in Figure 7 below. Hydrogel 1, Organogel 1, and Nanoemulsion 1 were randomly selected from the formulations that did not experience any problems as a result of stability studies and were used in the release study. When the release profile of propolis was examined, the release order was nanoemulsion, pure propolis, hydrogel, and organogel. After 12 h, an 82.58 ± 5.1% cumulative release was obtained from Nanoemulsion 1, 74.97 ± 3.49% from pure propolis, 57.31 ± 4.64% from Hydrogel 1, and 25.31 ± 3.78% from Organogel 1. The nanoemulsification of propolis in droplets caused an increase in the release, albeit slightly. The outer phase of the nanoemulsion transformed into a viscous base by entrapping the nanoemulsion into the gels. It is clear that this viscous gel base forms a barrier to the diffusion of propolis from the droplets. Therefore, it is usual for the release to be delayed in hydrogel and organogel. In addition, Carbopol 980 and other excipients in the organogel structure are thought to contribute to the delay of propolis release when the hydrogel is compared with the organogel. However, the concentrations, molecular weights, and viscosities of the polymers used in gel preparation also contribute significantly to the release [50]. In a study by Ghorpade et al., which included results similar to ours, gels were prepared using Carbopol 940, and high viscosity NaCMC, and a release study was performed with diffusion cells at pH 7.4. After 8 h of release, NaCMC gels prepared with a higher concentration (5%) showed higher release than Carbopol 940 gels prepared at 1% [51]. When the release profile of dexpanthenol was examined, the release order was pure dexpanthenol, nanoemulsion, and hydrogel = organogel. After 12 h, a 99.62 ± 1.46% cumulative release was obtained from pure dexpanthenol, 86.37 ± 3.25% from Nanoemulsion 1, 73.58 ± 4.46% from Hydrogel 1, and 73.43 ± 3.85% from Organogel 1. Dexpanthenol is a water-soluble substance that was present in all formulations in the external phase, not droplets. At the end of 12 h, ~100% release was achieved in pure dexpanthenol. On the other hand, in nanoemulsions, approximately 10% more release occurred than Hydrogel 1 and Organogel 1. Although dexpanthenol is in the outer phase in hydrogel and organogel, it can be said that polymer chains that cause viscous structure delay the release. Again, the oil droplets and surfactants present in the nanoemulsion slowed the release of dexpanthenol.

The release kinetics and mechanisms of propolis and dexpanthenol results are given in Table 7 below. In the evaluation made on the value of R^2^ for propolis Hydrogel 1, Organogel 1, and Nanoemulsion 1, the highest values in all formulations are seen in the zero-order model. For pure propolis, the highest value was seen in the Higuchi model. In the evaluation made on the value of R^2^ for dexpanthenol Hydrogel 1, Organogel 1, Nanoemulsion 1, and pure dexpanthenol, the highest values in all formulations were seen in the Higuchi model. The value of “*n*” is used to determine the drug release mechanism. If the *n* value is 0.5 or less, the release mechanism is a Fickian diffusion. If the *n* is 0.5 < *n* < 1, the release mechanism is a non-Fickian model denominated by anomalous transport. If the *n* value is 1, the release mechanism is a zero-order drug release or Case-II transport. In addition, for the values of *n* higher than 1, the release mechanism is super Case-II transport, which describes the influence of polymeric hydration and swelling on release for polymeric and swellable systems, and which can be related to matrix erosion for nonswellable systems [52,53]. Considering the *n* values, the release mechanism conforms to the Super Case-II Transport.

### 2.6. Antimicrobial Properties of Hydrogels and Organogels

Hydrogel 1, Organogel 1, and Nanoemulsion 1 were randomly selected from the formulations that did not experience any problems as a result of stability studies and were used in this study. To evaluate the antimicrobial susceptibility against *Staphylococcus aureus* ATCC 29213 (*S. aureus*), *Pseudomonas aeruginosa* ATCC 27853 (*P. aeruginosa*), *Escherichia coli* ATCC 25922 (*E. coli*), and *Staphylococcus epidermidis* ATCC 12228 (*S. epidermidis*), we determined the inhibition zone of pure propolis and pure dexpanthenol, and four gel formulations (blank hydrogel, Hydrogel 1, blank organogel, Organogel 1). The highest antimicrobial activity (22 mm in 100 µL) was detected in dexpanthenol against *S. aureus*. The antimicrobial activity of Hydrogel 1 in 50 µL and 100 µL concentrations was detected as 9 and 11 mm against *S. aureus*, respectively. On the other hand, the antimicrobial activity of 100 µL propolis was moderated (8 mm) against *S. epidermidis* and weaker (12 mm) than the Organogel 1. Blank hydrogel and blank organogel did not show antimicrobial activity against *S. aureus*, *P. aeruginosa*, *E. coli*, or *S. epidermidis* as expected. Of note, the antimicrobial susceptibility results were obtained by diluting the formulations in half (1:1 *v*/*v*, 0.25% propolis, and 2.5% dexpanthenol) with ultrapure water because of the high viscous structure of the hydrogels and organogels. Detailed antimicrobial activity results can be seen in Table 8. There is no antimicrobial activity study containing propolis and dexpanthenol in combination in the literature. However, there are studies in the literature that ethanolic propolis extract is sensitive to these bacteria [54,55,56,57]. We think this difference is caused by the oily extract we used in our study, which was standardized in terms of polyphenols.

### 2.7. Cytotoxic Properties of Hydrogels and Organogels

**Figure 8:**
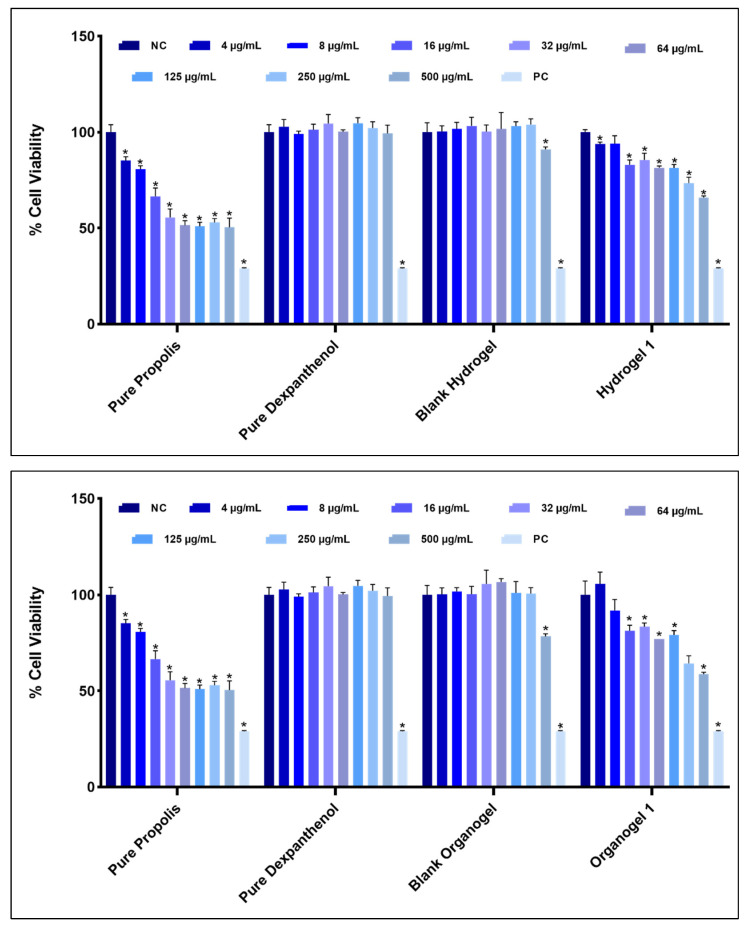
Cell viability of hydrogels, organogels, pure propolis, and pure dexpanthenol. Negative control (NC) cells were grown in the cell culture medium only. Positive control (PC) cells were treated with 1% Triton™ X-100. * Statistically significant compared to NC (*p* < 0.05).

**Figure 9:**
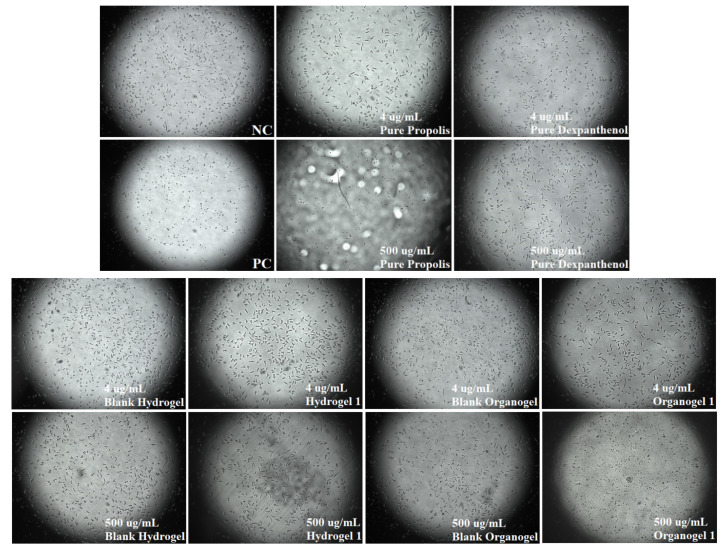
Cell morphology images of controls, pure propolis, pure dexpanthenol, hydrogels, and organogels.

Hydrogel 1, Organogel 1, and Nanoemulsion 1 were randomly selected from the formulations that did not experience any problems as a result of stability studies and were used in this study. CVDK-8 kit was used to assess the in vitro cytotoxic activity of pure propolis and pure dexpanthenol, blank hydrogel, Hydrogel 1, blank organogel, and Organogel 1 following the manufacturer’s guidelines. Figure 8 shows the differences in the percentages of cell viability of the pure propolis, pure dexpanthenol, hydrogels, and organogels that do not cause cytotoxicity to healthy human dermal fibroblasts. These data are important for determining ‘safe’ concentrations for future studies. In addition, Figure 9 includes cell morphology images. When cell viability was examined, the cytotoxic effect of blank hydrogel and blank organogel was not observed, except for the two highest doses. It is usual for the highest doses to show cytotoxic effects in cell culture studies. Doses of 250 and 500 µg/mL showed significant cytotoxicity over other doses. However, when the results of pure propolis, Hydrogel 1 and Organogel 1 were evaluated, these two high doses showed cytotoxic effect in all of them. In pure dexpanthenol, only 500 µg/mL showed cytotoxicity. Thus, our determinant was not these two high doses. This result was expected because dexpanthenol improves the stratum corneum and increases proliferation in fibroblasts [21,22]. Mencucci et al.’s human corneal and conjunctival cell culture study with MTT showed that dexpanthenol increased cell viability, similar to our results [58]. The 4 µg/mL dose of Organogel 1 is an important result to increase cell viability. There is no cell culture study with propolis oily extract in the literature. All doses of pure propolis (sunflower based extract) were found to be significantly cytotoxic. Since fixed oils cover the surface of the cells and prevent them from breathing, they may show a more cytotoxic effect. The reason why propolis is so cytotoxic is thought to be an oily extract. Further assessment should be done to confirm this data and explore its mechanism.

## 3. Conclusions

Recently, nanoemulsion-based gels have become very popular for dermal drug delivery, overcoming the disadvantages of conventional semi-solid drug forms. In this study, nanoemulsion-based hydrogels and organogels containing combined propolis and dexpanthenol were prepared and characterized. These hydrogels and organogels were compared in terms of their stability, antimicrobial, and cytotoxicity properties. Within the scope of characterization studies, organoleptic properties, drug content, morphology, pH, gel-sol conversion temperature, spreadability, viscosity, FT-IR, and release properties were evaluated in hydrogels and organogels. The characterization studies carried out were subjected to short-term stability evaluation at room temperature and refrigerator for 3 months, and satisfactory results were obtained. The release study successfully obtained an extended release for propolis and dexpanthenol. In the antimicrobial susceptibility study, Hydrogel 1 showed activity against *S. aureus*, while Organogel 1 showed activity against both *S. aureus* and *S. epidermidis*. In the cytotoxicity study against HDFa cells, both Hydrogel 1 and Organogel 1 were found to be nontoxic at low doses. As a result, the prepared hydrogels and organogels were successfully characterized, and satisfactory results were obtained in the antimicrobial susceptibility and cytotoxicity study. These hydrogels and organogels, which contain propolis and dexpanthenol in combination for the first time, are promising systems that can be used in wound and burn models in the future. More detailed studies should be done in the future.

## 4. Materials and Methods

### 4.1. Materials

Propolis oily extract (POLE G, B NATURAL) and dexpanthenol (Ph. Eur.) were kindly received as a gift from Barentz Turkey and BASF Turkey, respectively, and were used as active pharmaceutical ingredients. In addition, hydroxypropyl beta-cyclodextrin (HPβCD) and Carbopol 980 were kindly received as a gift from Barentz Turkey and İlko Pharmaceuticals, respectively. Tween 60, Span 80, NaOH, and KH_2_PO_4_ were purchased from Merck (Darmstadt, Germany). Ethanol, Tween 20, Span 20, and HCl were purchased from J. T. Baker (Gliwice, Poland), Merck (Darmstadt, Germany), Alfa Aesar (Kandel, Germany), and Isolab (Eschau, Germany), respectively. Sunflower oil was purchased from Hasyalçın Dış Tic. (Gaziantep, Turkey). Ultra-high viscosity NaCMC was purchased from Sigma (Amsterdam, The Netherlands). Ultrapure water (Direct-Q^®^ 3 UV, Merck Millipore, Darmstadt, Germany) was used in all water-requiring studies. The dialysis membrane (MWCO: 20 kD, Spectra/Por^®^Biotech) was purchased from Spectrum Laboratories, Inc., Gardena, CA, USA. Gentamicin was purchased from Oxoid (Hampshire, UK). For the cell culture study, adult Human Primary Dermal Fibroblast (HFDa) cells were purchased from ATCC (PCS-201-012™, Manassas, VA, USA). Gibco^TM^ DMEM and Gibco^TM^ FBS were purchased from ThermoFisher Scientific (Waltham, MA, USA). Penicillin/Streptomycin, L-glutamine, Trypsin/EDTA, DMSO and Triton^TM^ X-100 were purchased from Sigma (Darmstadt, Germany). PBS and cell viability detection kit 8 (CVDK-8) were purchased from Ecotech Biotechnology (Erzurum, Turkey). 

### 4.2. Development of Quantification Method for Propolis and Dexpanthenol

The propolis oily extract we used in our study is an standardized extract in terms of total polyphenols (>30 mg/mL, HPLC-ESI-MS) prepared by the dynamic multi-extraction method in sunflower oil in Italy. In our study, the oily propolis extract was then expressed as propolis. A stock solution of propolis containing polyphenol at a concentration of 100 μg/mL in ethanol was prepared by mixing on a multi-point magnetic stirrer (2mag, MIX 15 eco, Muenchen, Germany). Batches were prepared by dilution from this stock solution, and measurements were made in a UV-VIS spectrophotometer (Beckman Coulter DU 730, Brea, CA, USA). After the wavelength at which propolis gave maximum absorbance was found, the actual series (6 different concentrations, *n* = 6 of each) were prepared, and validation studies (accuracy, precision, LOD, LOQ, selectivity) were carried out with the calibration curve and equation [15].

A 1:3 ratio of dexpanthenol with HPβCD was prepared by mixing a stock solution of 200 μg/mL in ultrapure water for 20 min on a multi-point magnetic stirrer. An aqueous solution containing the same proportions of HPβCD as the blank solution was prepared. Batches were prepared by dilution from this stock solution, and measurements were made in a UV-VIS spectrophotometer. After the wavelength at which dexpanthenol gave maximum absorbance was found, the actual series (6 different concentrations, *n* = 6 of each) were prepared, and validation studies (accuracy, precision, LOD, LOQ, selectivity) were carried out with the calibration curve and equation [25].

### 4.3. Preparation of Nanoemulsions

The high-energy ultrasonication technique was used to prepare nanoemulsions containing propolis oily extract. First of all, propolis oily extract was homogenized with surfactants (such as Tweens and Spans). The dose of propolis was adjusted according to the amount of polyphenol in it, and it was in the form of 25 mg of polyphenol in 758.33 mg of propolis oily extract. Then, 250 mg of dexpanthenol was added to the water phase. The water phase was added to the oil phase, and ultrasonication (Bandelin Sonopuls HD 2070, Berlin, Germany) was applied for a certain time (100% amplitude, cycle 3), and nanoemulsions were formed (*n* = 6) [59]. Blank nanoemulsions were prepared according to the method described above without adding propolis and dexpanthenol.

### 4.4. Characterization of Nanoemulsions

The droplet size, polydispersity index, zeta potential, and conductivity of the freshly prepared nanoemulsions were determined by a Zetasizer (Malvern Zetasizer Nano ZSP, Cambridge, UK) in triplicate. Before the Zetasizer measurements, nanoemulsions were diluted at 1:10. This analysis was carried out at the East Anatolian High Technology Research and Application Center (DAYTAM) of Atatürk University. Apart from its conductivity, the dilution technique was also used for the type of determination of nanoemulsions. The prepared nanoemulsions were diluted at a 1:10 ratio with ultrapure water. In nanoemulsions where ultrapure water forms a homogeneous mixture and no phase separation was observed, the external phase was accepted as water [60]. The pH of the nanoemulsions was determined at room temperature with a pH meter (WTW inoLab, Weilheim, Germany) previously calibrated [61].

### 4.5. Preparation of Hydrogels and Organogels

#### 4.5.1. Preparation of Nanoemulsion Based Hydrogels

Ultra-high viscosity NaCMC was used in the preparation of hydrogels. First, 80 mg of NaCMC was left to swell on its own at room temperature with 2 g of distilled water. The nanoemulsions prepared into the fully swollen gels were added and mixed until homogeneous, and ultra-pure water was added until the total weight was 5 g (*n* = 6). The resulting hydrogels contain 0.5% propolis and 5% dexpanthenol. Blank hydrogels were prepared according to the method described above, with nanoemulsions prepared without adding propolis and dexpanthenol [62]. Hydrogels are coded according to the nanoemulsion numbers they contain. In other words, the hydrogel containing “Nanoemulsion 1” was coded as “Hydrogel 1” and the other hydrogels are coded by the same logic.

#### 4.5.2. Preparation of Nanoemulsion Based Organogels

Sunflower oil, Span 80, Tween 20, and Carbopol 980 were used to prepare organogels. First, 0.1 g of sunflower oil, 1:1 ratio of Span 80 and Tween 20, and 0.02 g Carbopol 980 were weighed and mixed until transparent. Then, the prepared nanoemulsions were added to this mixture and mixed until homogeneous, and pure water was added until the total weight was 5 g [63]. In the last step, NaOH solution was added dropwise for Carbopol 980 neutralization (*n* = 6) [64]. The resulting organogels contain 0.5% propolis and 5% dexpanthenol. Blank organogels were prepared according to the method described above, with nanoemulsions prepared without adding propolis and dexpanthenol. Organogels are coded according to the nanoemulsion numbers they contain. In other words, the organogel containing “Nanoemulsion 1” was coded as “Organogel 1” and the other organogels are coded by the same logic.

### 4.6. Characterization Studies of Hydrogels and Organogels

#### 4.6.1. Organoleptic Characteristics

In the organoleptic characterization study, the odors, colors, and phase separations of the hydrogels and organogels that were freshly prepared and kept at room temperature (24 ± 2 °C) and refrigerator (4 ± 2 °C) for 3 months were evaluated for stability. 

#### 4.6.2. TEM Images

The morphology of hydrogels and organogels was determined by transmission electron microscopy (TEM, Hitachi HighTech HT7700, Tokyo, Japan). For TEM imaging, hydrogels and organogels were dispersed in ultrapure water, and one drop of each diluted hydrogel and organogel was placed on 400-mesh carbon-coated copper grids. The grids were then dried at room temperature overnight. The TEM imaging was conducted at 120 kV. This analysis was carried out at the East Anatolian High Technology Research and Application Center (DAYTAM) of Atatürk University [65].

#### 4.6.3. Drug Contents

The amount of propolis and dexpanthenol in hydrogels and organogels was determined using the previously validated UV-VIS spectrophotometric method (*n* = 3). For this purpose, 3 mL of ethanol was added to each of the hydrogels and organogels, weighed in determined amounts, and mixed on a multi-point magnetic stirrer until they were completely clear. Afterward, the samples were centrifuged at 12,000 rpm for 15 min, and the absorbance values of the obtained supernatant were determined at 290 and 211 nm, respectively, in accordance with the calibration method for propolis and dexpanthenol separately. Hydrogel and organogel prepared without adding propolis and dexpanthenol were used as blanks [13,24]. In addition, within the scope of this study, the drug contents of hydrogels and organogels kept at room temperature and in the refrigerator for 3 months were evaluated in terms of stability.

#### 4.6.4. pH and Gel-Sol Transition Temperature

The pH of the freshly prepared hydrogels and organogels was determined at room temperature with a pH meter previously calibrated. In addition, within the scope of this study, the pH values of hydrogels and organogels kept at room temperature and in the refrigerator for 3 months were evaluated in terms of stability.

The gel-sol transition temperature study was carried out by modifying the method of Gopalan et al. [39]. The determined amounts of hydrogels and organogels, which were freshly prepared and kept at room temperature and in the refrigerator for 3 months for stability studies, were filled into the tubes and placed in a water bath at 37 °C with the help of a tube holder. Starting from 30 °C until 95 °C, the temperatures were increased by 5 degrees and waited for 5 min at each degree. At the end of each temperature, the tubes were examined by inverting one by one and the temperatures at which the hydrogels and organogels started to flow were determined.

#### 4.6.5. Spreadability and Viscosity Analysis

In the spreadability study, hydrogels and organogels, which were freshly prepared and kept at room temperature and refrigerator for 3 months, were evaluated (*n* = 3). 0.5 g of each hydrogel and organogel was weighed on a plastic plate (first plate) with a 1 cm diameter circle drawn in its center. The second plate was placed on the first plate, and a weight of 1 kg was placed on it and left for 5 min [39]. The spreadability of hydrogels and organogels was found by measuring the increasing diameter after 5 min and calculating with the equation given below [66]. The diameters obtained as a result of the spread were measured with the help of a manual caliper. S: Spreadability, m: weight of hydrogel/organogel (g), D: diameter of hydrogel/organogel (cm), t: holding time (second).

(1) $$S=\frac{m\;\times \;D}{t}$$

Hydrogels and organogels, which were freshly prepared and kept at room temperature and refrigerator for 3 months, were used in viscometer measurements (*n* = 3). Viscosity measurements were made in a rotational cone/plate viscometer (Brookfield DV2T-RV viscometer, Middleborough, MA, USA) using a CPA-40Z spindle for 1 min at 30 °C, 0.5 rpm, and a shear rate of 3750 1/s [67].

#### 4.6.6. FT-IR Analysis

FT-IR spectroscopy is an important technique used to elucidate the chemical composition and bond arrangements. Vibration frequencies of various bonds in the structure are measured with this technique, and information about the functional groups in the structure is obtained [68]. Whether there is a chemical interaction between propolis, dexpanthenol, excipients, hydrogels, and organogels, it was evaluated by examining the spectra taken in the range of 4000–400 cm^−1^ in the FT-IR spectrometer (Bruker VERTEX 70v, Billerica, MA, USA) [53].

#### 4.6.7. In Vitro Release and Release Kinetics

In vitro drug release studies were performed by the dialysis bag method to determine the propolis and dexpanthenol release (*n* = 3). For this purpose, a dialysis membrane (MWCO: 20 kD, Spectra/Por^®^ Biotech), pH 5.5 phosphate buffer, and a shaking water bath (Memmert WNB 14, Schwabach, Germany) were used. pH 5.5 phosphate buffer was prepared by adding HCl drop by drop into pH 5.8 phosphate buffer (USP 30/NF 25). The dialysis bags were incubated in the phosphate buffer for 30 min. In the study, the dose of propolis was adjusted to 5 mg and the dose of dexpanthenol to 50 mg, and the required amount of hydrogel, organogel, nanoemulsion, and pure propolis/dexpanthenol were weighed and placed in dialysis bags. Then, dialysis bags and 50 mL pH 5.5 phosphate buffer at 37 ± 0.5 °C were added to each amber bottle and shook at 50 rpm for 12 h in the water bath. At the specified time intervals (1, 2, 4, 6, 8, 12 h), 2 mL samples were taken from each bottle, and the same amount of fresh release medium was placed in each bottle to maintain the sink conditions. Afterward, the samples were centrifuged at 12,000 rpm for 15 min, and the absorbance values of the obtained supernatant were determined at 290 and 211 nm, respectively, in accordance with the calibration method for propolis and dexpanthenol, separately [69]. Hydrogel, organogel, and nanoemulsion prepared without adding propolis and dexpanthenol were used as blanks.

In vitro drug release results in pH 5.5 phosphate buffer were evaluated by a computer program in order to determine the best-fit release kinetic model of the hydrogels, organogels, nanoemulsions, and pure propolis/dexpanthenol. Whether formulations are compatible with zero-order, first-order, Higuchi, or Korsmeyer–Peppas kinetic models were determined by mathematical operations and formulas [70]. The release data obtained were fitted into the following equations. Q_t_: The released amount of propolis/dexpanthenol at time t, Q_0_: the initial amount of propolis/dexpanthenol, Q_∞_: the released amount of propolis/dexpanthenol at infinite time, k: the release constant of each model, *n*: the exponent, c: an intercept [71,72].

Zero-Order model: Q_t_/Q_∞_ = kt + c (2)

First-Order model: Q_t_ = Q_0_ + kt (3)

Higuchi model: Q_t_/Q_∞_ = kt^½^ + c (4)

Korsmeyer-Peppas model: Q_t_/Q_∞_ = kt*^n^* (5)

### 4.7. Antimicrobial Properties of Hydrogels and Organogels

The antimicrobial activity of hydrogel, organogel, blank hydrogel, blank organogel, propolis, and dexpanthenol was tested against *Staphylococcus aureus* ATCC 29213, *Pseudomonas aeruginosa* ATCC 27853, *Escherichia coli* ATCC 25922, and *Staphylococcus epidermidis* ATCC 12228 using agar-well diffusion test according to EUCAST [73]. The contents per well (4 mm diameter) were as follows: 25, 50, and 100 µL of formulations and pure propolis/dexpanthenol. The formulations with high viscosity were diluted half amount of sterile distilled water for transferring to the well on the agar plate. The gentamicin (10 µg) disc was used for the comparison of the antimicrobial susceptibility as a control. All of the disc diffusion tests were performed in triplicate with appropriate controls.

### 4.8. Cytotoxic Properties of Hydrogels and Organogels

HDFa cells were cultured in DMEM containing 10% FBS, 1% penicillin/streptomycin, and 1% L-glutamine and incubated at 37 °C in a humidified incubator (CelCulture^®^ CCL-170B-9, ESCO, Singapore) with 5% CO_2_. HDFa cells grown in cell culture flasks were removed from the attached surface using Trypsin/EDTA. The total cell number was calculated by the trypan blue method. Cells were seeded in 96-well plates in triplicate with 1.5 × 10^3^ cells per well. Samples prepared in the concentrations of 500, 250, 125, 64, 32, 16, 8, and 4 µg/mL were applied to the wells in triplicate, with a total volume of 100 µL in each well. Positive control cells were treated with 1% Triton™ X-100 and incubated for 24 h at 37 °C under 5% CO_2_. At the end of the incubation period, the medium in the wells was removed, and the medium containing 10% CVDK-8 solution was added to each well. Cells were incubated in the dark at 37 °C for up to 3 h in the incubator. Cell viability was determined by measuring optical density at 450 nm with an Epoch 2 Microplate Spectrophotometer (BioTek, Winooski, VT, USA). Changes in cell viability were calculated with reference to control groups.

### 4.9. Statistical Analysis

The statistical analysis between the samples was evaluated with a one-way analyses of variance (ANOVA) test (according to the homogeneity of the variances and the size of the population). Results at the *p* < 0.05 level were considered significant. All data in the cell culture study are given as mean ± standard deviation. All statistical analyzes were performed using the Student’s t-test, and *p*-values less than or equal to 0.05 were considered statistically significant.

## Figures and Tables

**Table 1:** Composition of propolis and dexpanthenol-loaded nanoemulsions (mg).

Formulation Code	Propolis	Dexpanthenol	Tween 60	Span 20	Tween 20	Span 80
Nanoemulsion 1	25	250	100	100	-	-
Nanoemulsion 2	25	250	200	100	-	-
Nanoemulsion 3	25	250	100	200	-	-
Nanoemulsion 4	25	250	-	-	100	100
Nanoemulsion 5	25	250	-	-	200	100
Nanoemulsion 6	25	250	-	-	100	200

**Table 2:** Characterization results of nanoemulsions.

Formulation Code	Droplet Size (nm) *	Polydispersity Index *	Zeta Potential (mV) *	Conductivity (µS/cm) *	pH
Nanoemulsion 1	211.2 ± 6.45	0.173 ± 0.015	−31.9 ± 0.52	17.00 ± 4.50	6.43
Nanoemulsion 2	185.5 ± 3.30	0.205 ± 0.006	−34.3 ± 0.31	15.40 ± 8.49	6.20
Nanoemulsion 3	166.0 ± 0.97	0.149 ± 0.002	−33.6 ± 1.55	12.10 ± 3.41	6.81
Nanoemulsion 4	221.6 ± 4.71	0.189 ± 0.014	−29.8 ± 0.45	16.90 ± 0.06	6.51
Nanoemulsion 5	203.3 ± 0.84	0.216 ± 0.005	−30.8 ± 0.83	12.00 ± 4.89	5.91
Nanoemulsion 6	219.8 ± 4.05	0.207 ± 0.009	−40.0 ± 1.42	7.20 ± 0.50	6.18

* Mean ± Standard deviation (SD).

**Table 3:** The phase separations and changes in colors after 3 months of storage.

Formulation Code	Phase Separation		Color	
	Room Temperature	Refrigerator Temperature	Room Temperature	Refrigerator Temperature
Hydrogel 1	-	-	Pale yellow to white	Pale yellow to white
Hydrogel 2	-	-	Pale yellow to white	Pale yellow to white
Hydrogel 3	-	-	Pale yellow to white	Pale yellow to white
Hydrogel 4	+	-	Pale yellow	Pale yellow to white
Hydrogel 5	+	-	Pale yellow	Pale yellow to white
Hydrogel 6	+	-	Pale yellow	Pale yellow to white
Organogel 1	-	-	Pale yellow to white	Pale yellow to white
Organogel 2	-	-	Pale yellow to white	Pale yellow to white
Organogel 3	-	-	Pale yellow to white	Pale yellow to white
Organogel 4	-	-	Pale yellow to white	Pale yellow to white
Organogel 5	-	-	Pale yellow to white	Pale yellow to white
Organogel 6	+	-	Pale yellow	Pale yellow to white

+: Phase separation occurred, -: No phase separation occurred.

**Table 4:** Drug contents of hydrogels and organogels (mean ± SD).

Formulation Code	Freshly Prepared		After 3 Months Room Temperature		After 3 Months Refrigerator Temperature	
	Propolis	Dexpanthenol	Propolis	Dexpanthenol	Propolis	Dexpanthenol
Hydrogel 1	102.06 ± 2.67	101.68 ± 0.68	102.00 ± 0.47	100.01 ± 0.63	102.23 ± 1.20	99.91 ± 0.85
Hydrogel 2	101.35 ± 3.13	100.18 ± 1.10	102.70 ± 0.47	99.04 ± 0.57	99.48 ± 1.30	99.69 ± 0.83
Hydrogel 3	100.24 ± 0.63	102.76 ± 1.87	99.54 ± 1.73	99.91 ± 0.98	102.06 ± 0.54	101.46 ± 1.31
Hydrogel 4	99.60 ± 1.17	101.26 ± 2.80	-	-	99.89 ± 0.54	99.99 ± 1.02
Hydrogel 5	100.05 ± 1.90	100.48 ± 4.42	-	-	102.29 ± 0.79	98.98 ± 1.49
Hydrogel 6	101.82 ± 0.98	99.76 ± 1.19	-	-	100.89 ± 1.54	101.36 ± 1.57
Organogel 1	99.89 ± 0.53	99.62 ± 1.46	102.82 ± 1.24	101.48 ± 0.95	101.12 ± 1.15	99.23 ± 0.82
Organogel 2	99.54 ± 2.10	98.15 ± 0.85	100.94 ± 1.10	99.78 ± 1.82	102.88 ± 2.03	99.55 ± 1.23
Organogel 3	100.59 ± 1.43	101.08 ± 1.36	103.47 ± 1.74	99.76 ± 1.74	100.71 ± 2.82	101.49 ± 1.51
Organogel 4	100.06 ± 1.69	99.98 ± 1.36	102.88 ± 0.30	101.12 ± 1.29	101.06 ± 1.86	98.92 ± 1.72
Organogel 5	99.89 ± 1.03	98.98 ± 1.02	102.70 ± 1.96	100.08 ± 1.67	102.18 ± 1.15	100.49 ± 1.26
Organogel 6	99.01 ± 1.84	100.05 ± 1.79	-	-	99.83 ± 1.00	100.42 ± 0.74

**Table 5:** The pH and gel-sol transition temperature results in hydrogels and organogels.

Formulation Code	pH			Gel-Sol Transition Temperature (°C)		
	Freshly Prepared	After 3 Months Room Temperature	After 3 Months Refrigerator Temperature	Freshly Prepared	After 3 Months Room Temperature	After 3 Months Refrigerator Temperature
Hydrogel 1	6.27	6.15	6.45	85	90	90
Hydrogel 2	6.25	6.17	6.05	90	90	90
Hydrogel 3	5.80	6.10	6.54	90	80	90
Hydrogel 4	5.99	6.13	6.16	85	-	90
Hydrogel 5	5.85	5.86	6.01	90	-	90
Hydrogel 6	6.20	5.95	5.76	90	-	90
Organogel 1	4.56	4.72	5.06	85	90	90
Organogel 2	4.51	4.52	4.74	85	95	85
Organogel 3	4.99	4.89	4.72	90	95	90
Organogel 4	4.62	4.78	4.63	85	95	95
Organogel 5	4.65	4.71	4.43	85	90	80
Organogel 6	4.52	4.54	4.35	90	-	85

**Table 6:** The spreadability and viscosity results of hydrogels and organogels (mean ± SD).

	Spreadability		g·cm/s Diameter ± SD		Viscosity (cP)		
Formulation Code	Freshly Prepared	After 3 Months Room Temperature		After 3 Months Refrigerator Temperature	Freshly Prepared	After 3 Months Room Temperature	After 3 Months Refrigerator Temperature
Hydrogel 1	15.66 ± 0.59 26.1 ± 1.0 mm	15.24 ± 0.08 25.4 ± 0.1 mm		19.23 ± 0.30 32.1 ± 0.5 mm	2528.7 ± 168.5	1706.7 ± 48.6	2585.7 ± 156.3
Hydrogel 2	24.42 ± 0.51 40.7 ± 0.8 mm	23.13 ± 0.55 38.6 ± 0.9 mm		17.31 ± 0.30 28.9 ± 0.5 mm	3479.0 ± 123.0	2533.3 ± 144.6	3425.7 ± 124.5
Hydrogel 3	15.00 ± 1.02 25.0 ± 1.7 mm	14.88 ± 0.25 24.8 ± 0.4 mm		21.63 ± 0.30 36.1 ± 0.5 mm	3690.7 ± 197.8	2792.7 ± 119.1	3816.7 ± 106.5
Hydrogel 4	24.99 ± 0.89 41.7 ± 1.5 mm	-		24.27 ± 0.38 40.5 ± 0.6 mm	3442.0 ± 163.7	-	3431.3 ± 104.6
Hydrogel 5	15.63 ± 1.32 26.1 ± 2.2 mm	-		15.12 ± 0.17 25.2 ± 0.3 mm	3350.7 ± 142.6	-	3291.7 ± 202.0
Hydrogel 6	23.88 ± 0.17 39.8 ± 0.3 mm	-		21.15 ± 0.13 35.3 ± 0.2 mm	3122.0 ± 250.6	-	3174.3 ± 114.7
Organogel 1	17.67 ± 0.13 29.5 ± 0.2 mm	14.64 ± 0.08 24.4 ± 0.1 mm		15.09 ± 0.13 25.2 ± 0.2 mm	1364.7 ± 100.2	1859.3 ± 92.8	1324.7 ± 56.8
Organogel 2	23.97 ± 1.15 40.0 ± 1.9 mm	30.93 ± 1.32 51.6 ± 2.2 mm		29.58 ± 0.51 49.3 ± 0.8 mm	1891.3 ± 71.9	1312.7 ± 127.5	1655.7 ± 129.0
Organogel 3	17.73 ± 0.21 29.6 ± 0.4 mm	14.70 ± 0.25 24.5 ± 0.4 mm		14.73 ± 0.98 24.6 ± 1.6 mm	1977.3 ± 172.7	996.0 ± 153.2	1827.3 ± 110.4
Organogel 4	23.43 ± 0.72 39.1 ± 1.2 mm	28.08 ± 2.21 46.8 ± 3.7 mm		28.08 ± 0.42 46.8 ± 0.7 mm	952.6 ± 135.1	143.9 ± 6.6	814.2 ± 125.1
Organogel 5	18.12 ± 0.08 30.2 ± 0.1 mm	14.97 ± 0.72 25.0 ± 1.2 mm		14.97 ± 0.04 25.0 ± 0.1 mm	1026.7 ± 121.1	344.5 ± 36.0	1085.0 ± 142.1
Organogel 6	23.76 ± 0.08 39.6 ± 0.1 mm	-		28.50 ± 0.59 47.5 ± 1.0 mm	1342.0 ± 115.4	-	1194.3 ± 92.9

**Table 7:** Release kinetics and mechanisms of propolis and dexpanthenol.

Formulation Code	Zero Order	First Order	Higuchi	Korsmeyer–Peppas		Release Mechanism
	R^2^	R^2^	R^2^	R^2^	*n*	
Propolis Hydrogel 1	0.989	0.644	0.987	0.886	1.512	Super Case-II Transport
Propolis Organogel 1	0.995	0.738	0.983	0.945	1.231	Super Case-II Transport
Propolis Nanoemulsion 1	0.995	0.589	0.970	0.826	1.749	Super Case-II Transport
Pure Propolis	0.987	0.604	0.994	0.868	1.802	Super Case-II Transport
Dexpanthenol Hydrogel 1	0.958	0.547	0.993	0.836	1.820	Super Case-II Transport
Dexpanthenol Organogel 1	0.944	0.512	0.992	0.808	1.813	Super Case-II Transport
Dexpanthenol Nanoemulsion 1	0.965	0.499	0.996	0.792	2.131	Super Case-II Transport
Pure Dexpanthenol	0.966	0.510	0.995	0.805	2.208	Super Case-II Transport

**Table 8:** Antimicrobial activity results from hydrogels, organogels, pure propolis, and pure dexpanthenol.

Formulation Code	*S. aureus*			*S. epidermidis*			*E. coli*			*P. aeruginosa*		
	25 µL	50 µL	100 µL	25 µL	50 µL	100 µL	25 µL	50 µL	100 µL	25 µL	50 µL	100 µL
Blank Hydrogel	-	-	-	-	-	-	-	-	-	-	-	-
Hydrogel 1	-	9	11	-	-	-	-	-	-	-	-	-
Blank Organogel	-	-	-	-	-	-	-	-	-	-	-	-
Organogel 1	-	-	8	8	12	12	-	-	-	-	-	-
Pure Propolis	-	-	-	-	-	8	-	-	-	-	-	-
Pure Dexpanthenol	12	20	22	-	-	-	-	-	-	-	-	-

## Data Availability

Not applicable.
